# ULF Pre-Seismic Geomagnetic Anomalous Signal Related to Mw8.1 Offshore Chiapas Earthquake, Mexico on 8 September 2017

**DOI:** 10.3390/e21010029

**Published:** 2019-01-03

**Authors:** Dragoș Armand Stănică, Dumitru Stănică

**Affiliations:** Department of Electromagnetism and Lithosphere Dynamics, Institute of Geodynamics of the Romanian Academy, R-020032 Bucharest, Romania

**Keywords:** ULF pre-seismic geomagnetic signal, Mw8.1 Chiapas-Mexico offshore earthquake, Teoloyucan and Tucson geomagnetic observatories, Cocos-North America subduction zone

## Abstract

In the last decade, the real time ground–based geomagnetic observations realized in correlation with the Vrancea seismicity in Romania, together with supplementary studies related to some earthquakes (Mw9.0 Tohoku, Japan on 11 March 2011 and Mw8.3 Coquimbo, Chile on 16 September 2015), enlarged our knowledge about the relationship between the pre-seismic anomalous phenomena and the final stage of the earthquake nucleation. To identify possible ultra-low-frequency (ULF) geomagnetic signals prior to the onset of an Mw8.1 earthquake, we retroactively analyzed the data collected on the interval 1 August–16 September 2017 at the Geomagnetic Observatories in Teoloyucan (TEO), Mexico and Tucson (TUC) USA, with the last taken as a reference. Daily mean distributions of the polarization parameter BPOL (geomagnetic polarization parameter) and standard deviation are obtained for both observatories using a fast Fourier transform (FFT) band-pass filtering in the ULF range (0.001–0.083 Hz). Further on, we investigated the singularity of the pre-seismic signal associated with an Mw8.1 earthquake and applied a statistical analysis based on a standardized random variable equation; results are presented as BPOL* time series on the interval 1–26 September. Finally, the hourly mean distribution, obtained as difference BPOL (TUC-TEO) on the interval 7–9 September emphasizes an anomalous signal with five hours before the onset of the Mw8.1 earthquake.

## 1. Introduction

Electromagnetic phenomena related to the seismic events have been extensively analyzed by different scientific groups, and a variety of the short-term pre-seismic anomalous signals covering a wide range of frequencies from DC to very high frequency (VHF) using ground-based and satellite observation techniques are detected [1]. In the first category, important contributions are given [2,3,4,5,6,7,8,9,10,11,12,13,14,15], while for the second one, the following papers are to be mentioned [16,17,18,19,20,21,22,23]. Furthermore, ionospheric perturbation related to seismicity have been detected with the use of very low frequency/low frequency (VLF/LF) signals, ionosondes, GPS Total Electron Content (TEC) observations, etc. [24,25]. Although, the origin of the ULF geomagnetic signal is not well-known yet, four possible generation mechanisms may be considered: (a) Magneto-hydrodynamic effect, which supposes that the conducting fluid flow, in the presence of a magnetic field, generates a secondary induced component [26]; (b) piezo-magnetic effect, based on the idea that a secondary magnetic field is induced by changes in ferromagnetic rocks magnetization due to an applied stress [27]; (c) electro-kinetic effect, based on electric currents flow at the interface solid–liquid boundaries, which in turn may generate a magnetic field [28]; and (d) piezo-stimulated current and current generated by charged dislocation [29]. A comprehensive analysis regarding the earthquake precursors associated with electromagnetic effects taking into account the place and magnitude of the earthquakes, date, type of the electromagnetic precursors detected, frequency range, instrumentation, method of detection with precursory time, effective distance from epicenter, and references are analyzed in detail [30]. The ULF geomagnetic pre-seismic signals associated with seven major earthquakes generated in Mexico during the interval of 1999–2001 are analyzed in detail [31]. Geomagnetic data collected from both ground-based and satellites observatories have an important role in the pre-seismic signal investigation. This means that gaining more accurate knowledge about the origin of the internal and external parts of the geomagnetic sources could give an accurate separation of the pre-seismic signal. Consequently, in this study, we used the data collected from the geomagnetic observatories in Teoloyucan, Mexico, and Tucson, USA. To differentiate the transient local anomalies associated with Mw8.1 earthquake by the internal and external parts of the geomagnetic field, we applied both the FFT Band-pass filtering (FFT-BPF) in ULF range (0.001–0.0083 Hz) and statistical analysis based on the standardized random variable equation, with the last observatory taken as a reference.

## 2. Methodology, Data Collection, Processing and Analyzing

A great Mw8.1 earthquake struck offshore of Chiapas, Mexico, on 8 September 2017 at 04:49 UTC located at geographic coordinates 15.02N and 93.81W, with a focal depth placed at 72 km, as was determined by the Euro-Mediterranean Seismic Centre (http://www.emsc-csem.org). This earthquake occurred on the subduction zone boundary between the oceanic Cocos and North America plates and was intensively felt in Guatemala City, about 1000 km away from Mexico City where important damages were observed at the international airport and some hospitals.

**Figure 1:**
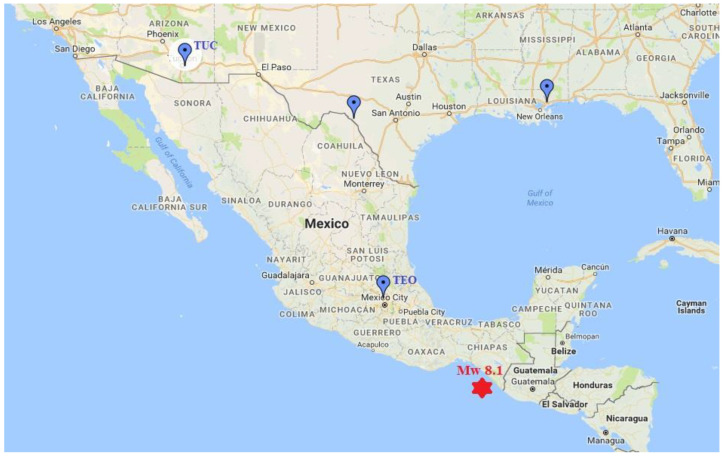
Map with the placements of the Mw8.1 earthquake (red star) and the Teoloyucan (TEO) and Tucson (TUC) geomagnetic observatories (blue marks).

To emphasize possible anomalous signals associated with this very large earthquake, in this paper we used the geomagnetic data (http://www.intermagnet.org) collected on the interval 1 August–26 September 2017 at the Geomagnetic Observatories in Teoloyucan (TEO), Mexico, and Tucson (TUC), USA, as shown in Figure 1. The following relations were used.

The polarization parameter (BPOL) is expressed as

BPOL(*f*) = *Bz*(*f*)/*SQRT*(*Bx*^2^(*f*) + *By*^2^(*f*)), (1)

where *Bx*, *By* and *Bz* are horizontal and vertical components of the geomagnetic field in µ*T*, *f* is frequency in (Hz) [32].

It is well known that for a 2-D geoelectric structure, the parameter (BPOL) may be associated with E-polarization mode, which describes electrical currents flowing parallel to strike (x direction) in terms of the electromagnetic field components *Ex*, *By*, *Bz*, as it is shown in Relation (2):

∂*Ex*/∂*y* = ∂*Bz*/∂*t* = *iwBz*; ∂*Ex*/∂*z* = ∂*By*/∂*t* = −*iwBy*; ∂*Bz*/∂*y* − ∂*By*/∂*z* = µσ*Ex*, (2)

where *w* is angular frequency (s^−1^), µ is magnetic permeability (VsA^−1^m^−1^ = Hm^−1^), σ is conductivity (Sm^−1^), and *Ex* is electric field parallel to strike (Vm^−1^).

In this particular case, the insulator (North American Plate)–conductor (Cocos Plate) boundary, extended through a 2-D geoelectric structure gives rise to an anomalous parameter (BPOL) that is orientated perpendicular to them and has magnitude proportional to the intensities of the current concentrations, which are, in turn, generated by the tectonic stress reached due to the Mw8.1 earthquake.

The long-range effect of the strain related to the pre-seismic geomagnetic signals, for which we used Relation (3), given by (Morgunov and Malzev, 2007) [33],

*R*(km) =10^0.5*M*−0.27^, (3)

where *R* is epicentral distance and *M* is earthquake magnitude.

According to Relation (3), the strain effect of the Mw8.1 Chiapas earthquake may be felt at *R* ≈ 6000 km, as in our particular case where the distances between the earthquake epicenter and both observatories are about 1000 km for TEO and 3000 km for TUC; the condition to identify a pre-seismic anomalous signal is fulfilled.

**Figure 2:**
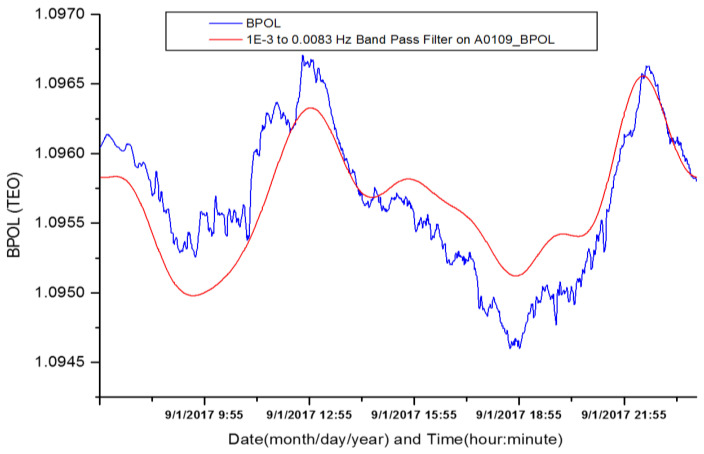
FFT (fast Fourier transform) Band-pass filtering (red line) applied on BPOL (geomagnetic polarization parameter) time series (blue line) for a time windows of 1024 samples recorded on 1 September 2017.

Further on, the daily mean distributions of the parameter BPOL with its standard deviation (SD) and BPOL* were obtained for TEO and TUC observatories, respectively, by using the following two procedures:

1. FFT Band-pass filtering (FFT-BPF) in the ULF range (0.001–0.0083 Hz) was applied to BPOL time series for two consecutive time windows of 1024 samples with about 40% overlapping on the 1440 dataset acquired every day, and an example for TEO observatory is presented in Figure 2 and Table 1;
2. Statistical analysis based on the standardized random variable equation was applied for the two particular cases:
  - to assess the singularity of the pre-seismic anomalous signal, related to the Mw8.1 earthquake, observed on the daily mean distributions of the BPOL (TEO) and BOPL (TUC), by using following relation:
    BPOL* = (*X* − *Y*)/*Z*, (4)
    where
    - *X* is the value of the of BPOL for a particular day, starting with 1 September 2017 and ending on 26 September 2017;
    - *Y* is 30 days running average of BPOL for consecutive days before a particular day;
    - *Z* is 30 days running average of SD obtained for 30 consecutive days before a particular day;
    - BPOL* emphasizes the threshold for anomaly using SD;
  - to differentiate the transient local anomalies associated with an Mw8.1 earthquake by the internal and external parts of the geomagnetic field, taking the Geomagnetic Observatory (TUC) as reference, we used the following relation:
    BPOL*(TUC-TEO) = (*A* − *B*)/*W*, (5)
    where
    - *A* is the value of the (BPOL TUC-BPOL TEO) for a particular day, starting with 1 September and ending with 26 September 2017;
    - *B* is 30 days running average of (BPOL TUC-BPOL TEO) before the particular day;
    - *W* is 30 days running average of (SD TUC-SD TEO) before the particular day;
    - BPOL*(TUC-TEO) time series emphasizing the threshold for anomaly using SD.

## 3. Results

Based on Relations (1), (4) and (5), in the next three sections (Section 3.1, Section 3.2 and Section 3.3), we present the daily mean distributions of the geomagnetic parameters BPOL, BPOL* and BPOL* (TUC-TEO) carried out for the geomagnetic observatories TEO and TUC for the two time intervals: 1 August–26 September 2017 and 1–26 September 2017. Additionally, to improve the quality of information related to the Mw8.1 earthquake, we analyzed the three-day interval (7–9 September) of hourly mean distribution of the BPOL (TUC-TEO).

### 3.1. BPOL and BPOL* Distributions Carried Out at the TEO Observatory Using Relations (1) and (4) 

**Figure 3:**
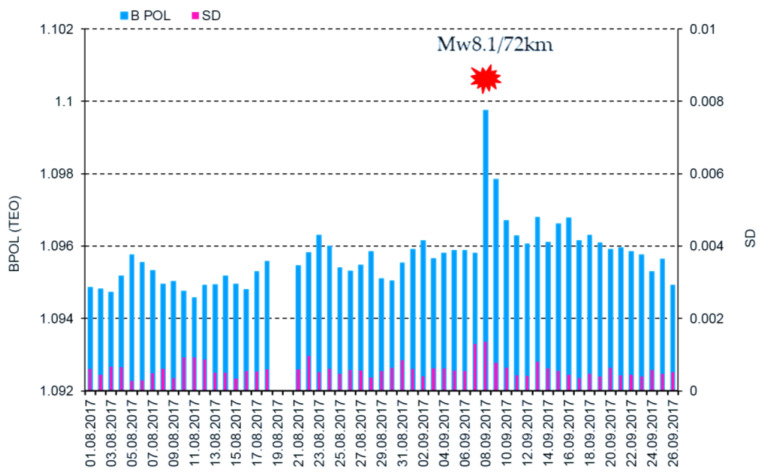
Daily mean distribution of the parameter BPOL (TEO) and SD on the interval 1 August–26 September 2017. Blue vertical bar is BPOL; red vertical bar is SD; and red star is the Mw8.1 earthquake.

**Figure 4:**
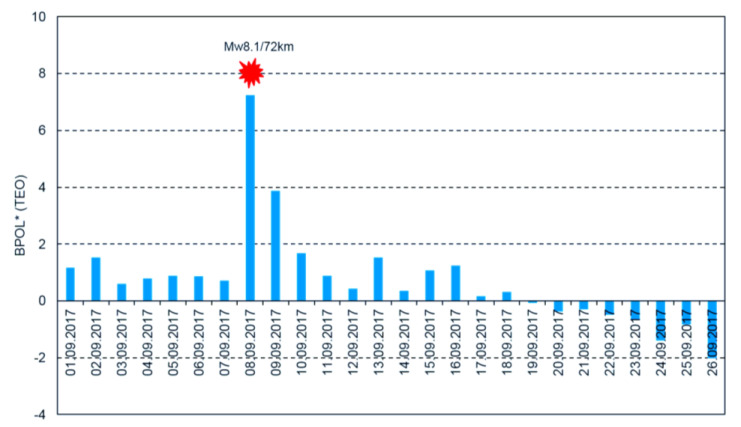
Daily mean distribution of the parameter BPOL* (TEO) on the interval 1–26 September 2017. Blue vertical bar is BPOL* (TEO); red star is the earthquake; and ratio Mw8.1/72 km is the earthquake magnitude/hypocenter depth, in kilometers.

To have a comprehensive view regarding the applied methodology, the daily mean distributions of the BPOL and BPOL* related to the major Mw8.1 earthquake are presented in Figure 3 and Figure 4.

### 3.2. BPOL and BPOL* Distributions Carried Out at the TUC Observatory Using Relations (1) and (4) 

**Figure 5:**
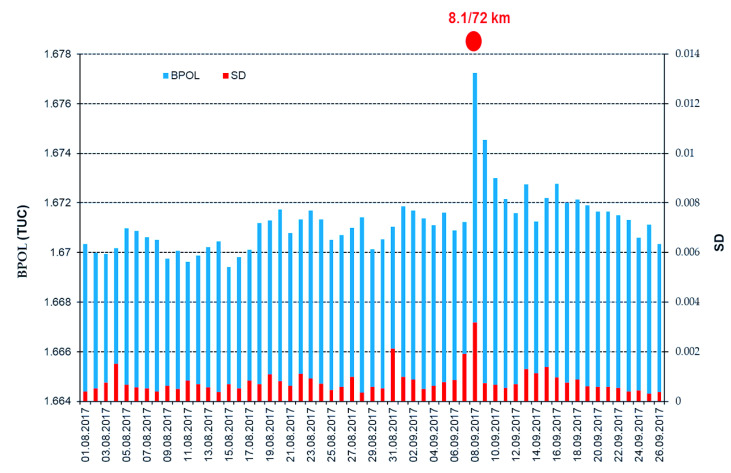
Daily mean distribution of the parameter BPOL (TEO) and SD on the interval 1 August–26 September 2017. Blue vertical bar is BPOL; red vertical bar is SD; red full circle is the earthquake; and ratio 8.1/72 km is the earthquake magnitude/hypocenter depth, in kilometers.

**Figure 6:**
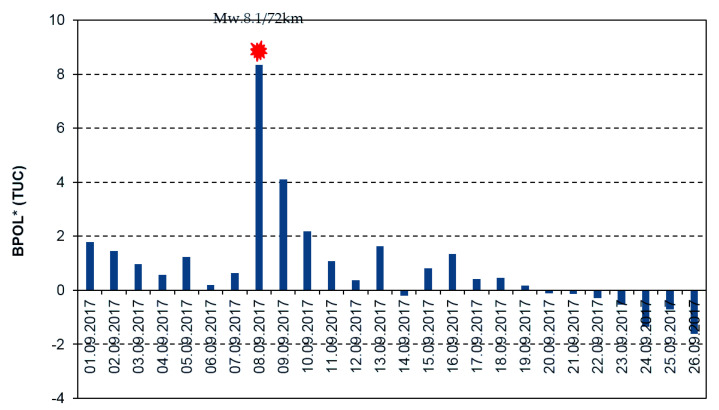
Daily mean distribution of the parameter BPOL* (TUC) on the interval 1–26 September 2017. Blue vertical bar is BPOL*(TUC); red full star is the earthquake; and ratio Mw8.1/72 km is the earthquake magnitude/hypocenter depth.

In Figure 5 and Figure 6, the daily mean distributions of the geomagnetic parameters BPOL (TUC) and BPOL* (TUC), in correlation with Mw8.1 earthquake, are shown.

### 3.3. BPOL* (TUC-TEO) Time Series and BPOL (TUC-TEO) Hourly Mean Distribution

**Figure 7:**
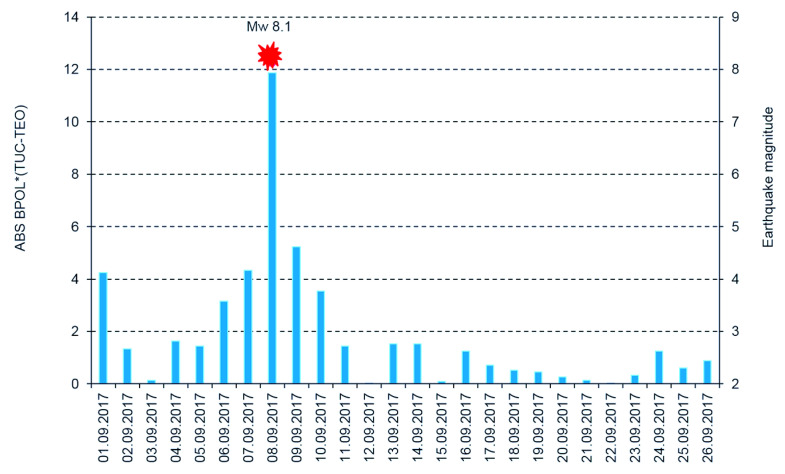
ABS BPOL* (TUC-TEO) time series and earthquake magnitude carried out on the interval 1–26 September 2017. ABS is absolute value; blue vertical bar is BPOL* (TUC-TEO); and red star is the Mw8.1 earthquake.

**Figure 8:**
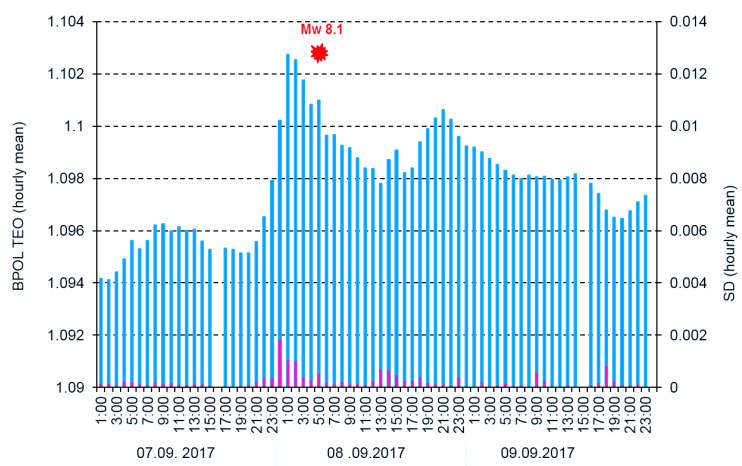
Hourly mean distribution of the parameter BPOL (TEO) and SD on the interval 7–9 September 2017. Blue vertical bar is BPOL TEO; red vertical bar is SD; and red full star is the Mw8.1 earthquake.

**Figure 9:**
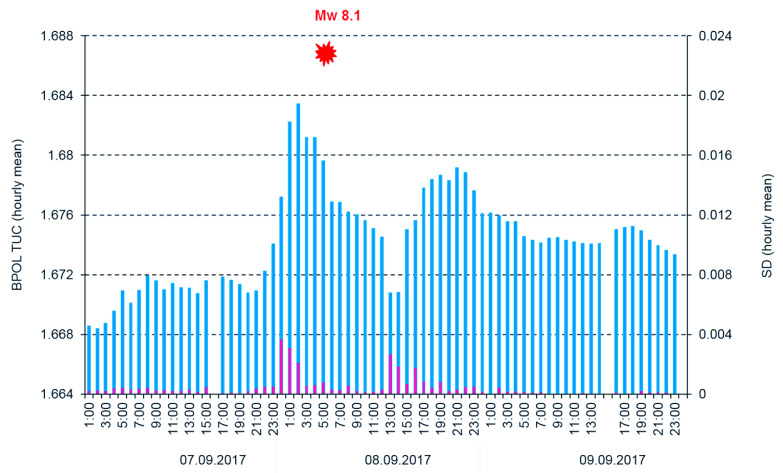
Hourly mean distribution of the parameter BPOL (TUC) and SD on the interval 7–9 September 2017. Blue vertical bar is BPOL (TEO); red vertical bar is SD; and red full star is the Mw8.1 earthquake.

**Figure 10:**
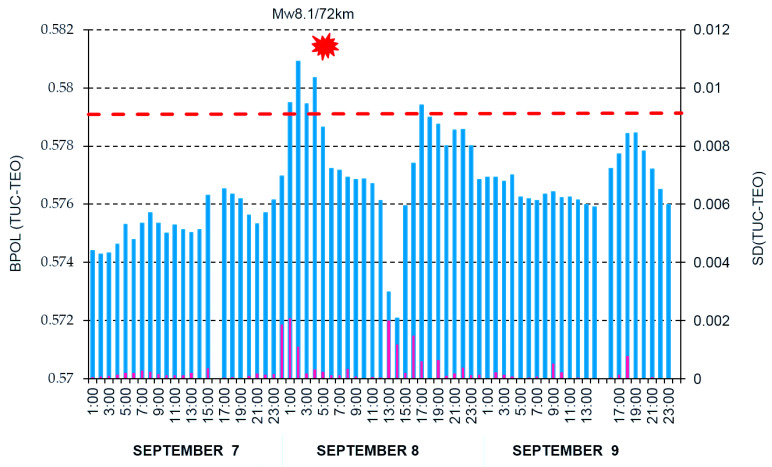
Hourly mean distribution of the parameter BPOL (TUC-TEO) and SD (TUC-TEO) on the interval 7–9 September 2017. Blue vertical bar is BPOL (TUC-TEO); red vertical bar is SD (TUC-TEO); red dashed line represents threshold for anomaly using SD; red full star is the earthquake; and Mw8.1/72 km is the magnitude/hypocentre depth.

To differentiate the pre-seismic anomalous signals related to Mw8.1 earthquake by the external part of the geomagnetic field, we applied two procedures:

1. First one based on Relation (5) when we used the Geomagnetic Observatory (TUC) as reference and result is shown in Figure 7;
2. Second one which uses the BPOL (TUC-TEO) as hourly mean distribution, obtained as amplitude differences between BPOL (TUC) and BPOL (TEO) for three consecutive days: 7–9 September 2017 (see Figure 8, Figure 9 and Figure 10).

## 4. Discussion and Conclusions

In order to emphasize possible interrelation between the pre-seismic geomagnetic signature and the Mw8.1 Chiapas earthquake, in this paper we have investigated the ULF geomagnetic data recorded at the Teoloyucan and Tucson geomagnetic observatories on the interval August–September 2017. Thus, the daily mean distributions of the BPOL (TEO) and BPOL (TUC), presented in Figure 3 and Figure 5, were analyzed for the interval 1 August–26 September 2017, both emphasizing the 8 September very high amplitudes (1.0988 for TEO and 1.6772 for TUC) which are associated with Mw8 earthquakes, suggesting the existence of a co-seismic effect. Furthermore, we analyzed the BPOL* (TEO) and BPOL* (TUC) time series obtained on the interval 1–26 September by using a statistical analysis based on Relation 4 and the results emphasize

- an anomalous interval of BPOL* (TEO), extended on 8 and 9 September, with values of 7.3 and 3.8, which are considered to be co- and post-seismic effects related to both Mw8.1 earthquake and the after-shocks with magnitude higher than 5, as shown in Figure 4; and
- BPOL* (TUC) time series with an anomalous interval observed on 8 and 9 September, having values of 8.3 and 4.1, that could be associated with the Mw8.1 earthquake and the after-shocks, both emphasizing co- and post-seismic effects; see Figure 6.

All the above information has stress; however, serious difficulties exist in detecting a pre-seismic geomagnetic signature by using individual observation sites. Therefore, simultaneous analysis of the geomagnetic data obtained in the two observatories (TEO and TUC), with the last one taken as reference, offer the opportunity to separate the seismogenic signals from the non-seismic external geomagnetic variations. The results are summarized in Section 3.3, Figure 7 and Figure 10, and as follows:

- A very clear anomaly of a maximum, extended between 6–10 September 10, with an apex of about 11.862 on 8 September, is detected on the ABS BPOL* (TUC-TEO) time series carried out on 1–26 September 2017 by using Relation (5) and is shown in Figure 7.
- The new time series of BPOL (TUC-TEO), obtained as hourly mean distribution on the interval 7–9 September, indicates a pre-seismic anomaly, placed between hours 1 and 4 on 8 September; see Figure 10.
- The anomalous behavior, manifested on the both distributions ABS*BPOL (TUC-TEO) and BPOL (TUC-TEO), indicate that their variability is not random, being significant and reliable pre-seismic signals associated with Mw8.1 earthquake. The last one, with magnitude higher than 4∙SD, was triggered with about five hours prior to the onset of the Mw8.1 seismic event, on 8 September 2017; see Figure 10.
- The anomalies are observed in the BPOL (TUC-TEO) distribution on 8 and 9 September, after the main shock are associated with the superposition effects of a lot of after-shocks with magnitudes higher than 5.

Finally, all mentioned results could offer opportunities to develop new tools for early detection of anomalies related to the major earthquakes. However, in terms of practical application, both BPOL and BPOL* parameters, as well as Bzn and Bzn* [34], are used in Romania for real time monitoring of the pre-seismic geomagnetic signals related to the Vrancea earthquakes.

The present analyzing was made, as mentioned, during the period 1 August–26 September 2017. As an outlook for the future, however, the present analysis should be extended in a future publication by starting it from the beginning of June 2017 because in two recent publications, i.e., References [35,36], precursory variations of the entropy change of seismicity in the Chiapas region in Mexico under time reversal were observed on 14 June 2017.

## Figures and Tables

**Table 1:** BPOL, BPOL (FFT-BPF) time series for 23 minutes on September 1, 2017, BPOL mean and BPOL(FFT-BPF) mean are daily averaged values and 1024 averaged values, respectively.

Time (h:mm)	BPOL	BPOL Mean	SD	BPOL FFT-BPF	BPOL (FFT-BPF) Mean	SD
0:00	1.09647	1.09591	0.00061	1.09576	1.09610	0.00039
0:01	1.09648			1.09579		
0:02	1.09651			1.09581		
0:03	1.09652			1.09584		
0:04	1.09654			1.09586		
0:05	1.09654			1.09589		
0:06	1.09653			1.09591		
0:07	1.09653			1.09594		
0:08	1.09655			1.09596		
0:09	1.09658			1.09599		
0:10	1.09662			1.09601		
0:11	1.09665			1.09604		
0:12	1.09666			1.09606		
0:13	1.09669			1.09609		
0:14	1.09670			1.09611		
0:15	1.09672			1.09614		
0:16	1.09675			1.09616		
0:17	1.09677			1.09619		
0:18	1.09681			1.09621		
0:19	1.09682			1.09624		
0:20	1.09684			1.09626		
0:21	1.09686			1.09628		
0:22	1.09688			1.09633		

## References

[1] Hayakawa M. (2015). Earthquake Prediction by Radio Techniques.

[2] Gokhberg M., Yoshino T., Morgunov V. (1982). Results of recording operative electromagnetic earthquake precursor in Japan. Phys. Solid Earth.

[3] Hayakawa M., Fujinawa Y. (1994). Electromagnetic Phenomena Related to the Earthquake Prediction.

[4] Huang Q. (2011). Retrospective investigation of geophysical data possibly associated with the Ms8.0 Wenchuan earthquake in Sichuan, China. J. Asian Earth Sci..

[5] Korytenko Y.A., Matiashvili T.G., Voronov P.M., Korytenko E.A., Hayakawa M., Fujinawa A.Y. (1994). Observation of electromagnetic ultra-low frequency lithospheric emission in the Caucasian seismically active zone and their connection with earthquakes. Electromagnetic Phenomena Related to Earthquake Prediction.

[6] Nagao T., Enomoto Y., Fujinawa Y., Hata M., Hayakawa M., Huang Q., Izutsu J., Kushida Y., Maeda K., Oike K. (2002). Electromagnetic anomalies associated with 1995 Kobe earthquake. J. Geodyn..

[7] Varotsos P., Sarlis N., Skordas E. (2003). Electric Field that “Arrive” before the Time Derivative of the Magnetic Field prior to Major Earthquake. Phys. Rev. Lett..

[8] Varotsos P., Sarlis N., Skordas E., Lazaridou M. (2007). Electric pulses some minutes before earthquake occurrence. Appl. Phys. Lett..

[9] Biagi P.F., Maggipinto T., Righetti F., Loiacono D., Schiavulli L., Ligonzo T., Ermini A., Moldovan I.A., Moldovan A.S., Buyuksarac A. (2011). The European VLF/LF radio network to search for earthquake precursors: Setting up and natural/man-made disturbances. Nat. Hazards Earth Syst. Sci..

[10] Sarlis N., Skordas E., Varotsos P., Nagao T., Kamogawa M., Tanaka H., Uyeda S. (2013). Minimum of the order parameter fluctuations of seismicity before major earthquake in Japan. Proc. Natl. Acad. Sci. USA.

[11] Varotsos P.A., Sarlis N.V., Skordas E.S., Lazaridou M.S. (2013). Seismic Electric Signals: An additionally fact showing their physical interconnection with seismicity. Tectonophysics.

[12] Stanica D., Stanica D.A. (2011). Anomalous pre-seismic behaviour of the electromagnetic normalized functions related to the intermediate depth earthquakes occurred in Vrancea zone, Romania. Nat. Hazards Earth Syst. Sci..

[13] Stanica D., Stanica D.A., D’Amico S. (2012). Earthquakes Precursors. Earthquake Research and Analysis, Statistical Studies, Observations and Planning.

[14] Stanica D.A., Stanica D., Vladimirescu N. (2015). Long-range anomalous electromagnetic effect related to M9 Great Tohoku earthquake. Earth Sci..

[15] Uyeda S., Nagao T., Kakogawa M. (2011). Earthquake Prediction and Precursor. Encyclopedia of Solid Earth Geophysics.

[16] Tramutoli V., Cuomo V., Filizzola C., Pergola N., Pietrapertosa C. (2005). Assessing the potential of thermal infrared satellite surveys for monitoring seismically active areas. The case of Kocaeli (Yzmit) earthquake, August 17th, 1999. Remote Sens. Environ..

[17] Ouzounov D., Freund F. (2004). Mid–infrared emission prior to strong earthquakes analyzed by remote sensing data. Adv. Space Res..

[18] Ouzounov D., Liu D., Chunli K., Cevone G., Kafatos M., Taylor P. (2007). Outgoing long wave radiation variability from IR satellite data prior to major earthquakes. Tectonophysics.

[19] Pulinets S.A., Ouzounov D., Karelin A.V., Boyarchuk K.A., Pokhmelnykh L.A. (2006). The physical nature of the thermal anomalies observed before strong earthquakes. Phys. Chem. Earth.

[20] Błęcki J., Parrot M., Wronowski R. (2010). Studies of the Electromagnetic Field Variations in ELF Frequency Range Registered by DEMETER Over the Sichuan Region Prior to the 12 May 2008 Earthquake. Int. J. Remote Sens..

[21] Błęcki J., Parrot M., Wronowski R. (2011). Plasma turbulence in the ionosphere prior to earthquakes, some remarks on the DEMETER registrations. J. Asian Earth Sci..

[22] Hayakawa M., Hobara Y., Ohta K., Hattori K. (2011). The Ultra-Low-Frequency Magnetic Disturbances Associated with Earthquakes. Earthq. Sci..

[23] Parrot M., Berthelier J.J., Blecki J., Brochot J.Y., Hobara Y., Lagoutte D., Lebreton J.P., Němec F., Onishi T., Pinçon J.L. (2015). Unexpected events recorded by the ionospheric satellite DEMETER. Surv. Geophys..

[24] Hayakawa M. (2011). Probing the lower ionospheric perturbations associated with earthquakes by means of subionospheric VLF/LF propagation. Earthq. Sci..

[25] Liu J.Y., Hayakawa M. (2009). Earthquake precursors in ionospheric F-region. Electromagnetic Phenomena Associated with Earthquakes.

[26] Sasai Y. (1991). Tectonomagnetic modeling on the basis of the linear Piezomagnetic effect. Bull. Earthq. Res. Inst. Univ. Tokyo.

[27] Fitterman D.V. (1978). Electrokinetic and magnetic anomalies associated with dilatant regions in a layered Earth. J. Geophys. Res..

[28] Fitterman D.V. (1979). Theory of electrokinetic-magnetic anomalies in a faulted half-space. J. Geophys. Res..

[29] Varotsos P., Alexopoulos K., Nomicos K., Lazaridou M. (1986). Earthquake prediction and electric signal. Nature.

[30] Petraki E., Nikolopoulos D., Nomicos K., Stonham J., Cantzos D., Yannakopoulos P., Kottou S. (2015). Electromagnetic Pre-earthquake Precursors: Mechanisms, Data and Models-A Review. J. Earth Sci. Clim. Chang..

[31] Kotsarenko A., Perez Enriquez R., Lopez Cruz-Abeyro J.A., Koshevaya S., Grimalsky S., Yutsis I., Kremenetsky I. (2007). ULF geomagnetic anomalies of possible seismogenic origin observed at Teoloyucan station, México, in 1999–2001: Intermediate and Short-Time Analysis. Tectonophysics.

[32] Hayakawa M., Kawate R., Molchanov O.A., Yumoto K. (1996). Results of ultra- Low-frequency magnetic field measurements during the Guam earthquake of 8 August 1993. Geophys. Res. Lett..

[33] Morgunov V., Malzev S. (2007). A multiple fracture model of pre-seismic electromagnetic phenomena. Tectonophysics.

[34] Stanica D.A., Stanica D., Błęcki J., Ernst T., Jozwiak W., Słomiński J. (2018). Pre-seismic geomagnetic and ionosphere signature related to the Mw5.7 earthquake occurred in Vrancea zone on September 24, 2016. Acta Geophys..

[35] Ramirez-Rojas A., Flores-Marquez E.L., Sarlis N.V., Varotsos P.A. (2018). The Complexity Measurements Associated with the Fluctuations of the Entropy in Natural Time before the Deadly Mexico M8.2 Earthquake on 7 September 2017. Entropy.

[36] Sarlis N.V., Skordas E.S., Varotsos P.A., Ramirez-Rojas A., Flores-Marquez E.L. (2018). Natural time analysis: On the deadly Mexico M8.2 earthquake on & September 2017. Physica A.

